# Mindfulness and trauma recovery among internally displaced individuals: the mediating role of distress tolerance in a cross-sectional mediation model

**DOI:** 10.3389/fpsyg.2025.1677965

**Published:** 2025-09-24

**Authors:** Aamer Aldbyani, Afnan Alhimaidi, Zhang Chuanxia

**Affiliations:** ^1^Department of General Education, Shandong Xiehe University, Jinan, China; ^2^Department of Psychology, Princess Nourah Bint Abdulrahman University, Riyadh, Saudi Arabia

**Keywords:** mindfulness, distress tolerance, trauma recovery, internally displaced individuals, mediation

## Abstract

**Background:**

Mindfulness has gained increasing attention for its role in fostering emotional regulation and psychological resilience. In contexts of displacement and trauma, mindfulness may be associated with improved adaptive coping and recovery. This cross-sectional study investigated the relationships among mindfulness, distress tolerance, and trauma recovery in internally displaced individuals, with a focus on the mediating role of distress tolerance.

**Methods:**

A sample of 321 Yemeni internally displaced persons completed the Five Facet Mindfulness Questionnaire (FFMQ), the Distress Tolerance Scale (DTS), and the Posttraumatic Diagnostic Scale for DSM-5 (PDS-5).

**Results:**

Analysis revealed that all facets of mindfulness—observing, describing, acting with awareness, non-reactivity, and non-judgment—were positively associated with both distress tolerance and trauma recovery. Mediation models indicated that distress tolerance partially mediated the relationship between mindfulness and trauma recovery.

**Conclusion:**

These findings indicate the potential value of including mindfulness and distress-tolerance components in interventions for internally displaced populations.

## 1 Introduction

Mindfulness, a central concept in contemporary positive psychology, focuses on enhancing positive human functioning and has been increasingly applied beyond the realm of psychopathology. In the context of trauma, it is theorized to denote a state of awareness, encompassing a set of cognitive and emotional capacities, as well as a trait-like disposition (Randal et al., 2015). Mindfulness involves the deliberate and sustained attention to present-moment experiences in a nonjudgmental and objective manner (Garland and Howard, 2013). It can be cultivated through structured training programs and has been widely examined across diverse populations, including students, healthcare professionals, athletes, and parents (Aldbyani, 2023; Birrer et al., 2012; Brown et al., 2007; Kabat-Zinn, 2003). Individuals with higher levels of mindfulness tend to exhibit increased empathy, self-acceptance, and emotional balance, as well as healthier lifestyle behaviors (Burgoon et al., 2000; Feltman et al., 2009). Furthermore, mindfulness has been linked to lower levels of anxiety, depression, psychological distress, and stress (Alzahrani et al., 2020; Kabat-Zinn and Kabat-Zinn, 2021).

Beyond its general psychological benefits, mindfulness may contribute to aspects of trauma recovery. Trauma refers to experiences that exceed an individual’s coping capacity and disrupt their psychological functioning (Wilson et al., 2001). Several studies have indicated that mindfulness may facilitate trauma recovery by counteracting avoidance-based coping strategies commonly observed among trauma survivors. Individuals with low mindfulness often engage in psychological inflexibility and maladaptive behaviors, such as emotional suppression and experiential avoidance, which may exacerbate trauma-related symptoms (Follette et al., 2006; Wegner and Zanakos, 1994; Aizik-Reebs et al., 2021). In contrast, mindful awareness encourages acceptance and present-focused processing, reducing avoidance tendencies and supporting psychological healing (Palm et al., 2004; Hayes and Gifford, 1997). These findings provide empirical support for the therapeutic potential of mindfulness in alleviating trauma-related distress and promoting adaptive functioning.

Defined as intentional, present-moment awareness and nonjudgmental acceptance (Kabat-Zinn, 2003; McDonough and Lemon, 2018), mindfulness fosters emotional regulation and psychological flexibility. Research demonstrates that individuals who adopt a mindful orientation are more capable of observing their thoughts and feelings without becoming overwhelmed. As such, they are more likely to respond adaptively to distressing experiences.

While mindfulness appears to support trauma recovery directly, its influence may also operate indirectly through distress tolerance. Distress tolerance refers to one’s ability to endure aversive emotional states (Simons and Gaher, 2005). Evidence suggests that mindfulness enhances this capacity. Brief mindfulness-based interventions have been shown to reduce pain perception and improve behavioral persistence in tasks involving psychological discomfort (Liu et al., 2013; Feldman et al., 2014). Moreover, individuals who engage in mindful self-reflection report higher distress endurance compared to those who rely on ruminative or avoidant strategies (Sauer and Baer, 2012).

Improved distress tolerance is associated with better psychological outcomes following trauma. It contributes to emotional regulation and adaptive coping, thereby mitigating symptoms of posttraumatic stress and enhancing one’s ability to manage emotional responses under pressure (Fetzner et al., 2014). Accordingly, distress tolerance may serve as a psychological buffer that mediates the relationship between mindfulness and trauma recovery. Building on theoretical and empirical foundations that link mindfulness to emotional regulation, distress tolerance emerges as a plausible mechanism through which present-moment awareness fosters resilience and recovery.

In the context of Yemen—where protracted conflict has led to widespread displacement and chronic stress—mindfulness may be associated with better trauma-recovery indicators. This study builds on existing literature by investigating the direct and indirect effects of mindfulness on trauma recovery among internally displaced individuals in Yemen, with distress tolerance examined as a potential mediator. By focusing on trait (dispositional) mindfulness and its five dimensions, the study aims to clarify how present-moment awareness contributes to recovery in a high-stress, displacement-affected population. In light of the humanitarian crisis and limited access to mental health services, mindfulness may serve as a low-cost, scalable intervention to enhance emotional resilience and coping in this vulnerable group.

Although mindfulness has received increasing global attention for its psychological benefits, empirical research remains limited regarding its interaction with distress tolerance in the context of trauma recovery among displaced populations. Few studies have examined the simultaneous influence of all five facets of mindfulness on trauma-related outcomes using Mediation modelling approaches, particularly in conflict-affected settings such as Yemen.

Building upon prior research, the current study formulates the following hypotheses:

*H1: Mindfulness positively correlates with distress tolerance and trauma recovery among internally displaced individuals.*

*H2: Distress tolerance mediates the association of mindfulness and trauma recovery among internally displaced individuals.*

## 2 Method

### 2.1 Participants

A total of 321 Yemeni internally displaced persons (230 males and 91 females) voluntarily participated in this study. Participants’ ages ranged from 24 to 54 years (Mage = 34.13). Recruitment was conducted online via Facebook between March and May 2025. Inclusion criteria were: being Yemeni, currently displaced within Yemen due to conflict, aged 18 years or older, and fluent in Arabic. Individuals who did not self-identify as internally displaced persons or who provided incomplete responses were excluded. To validate participant status (e.g., current governorate of residence and original place of origin), responses inconsistent with IDP status were removed. Informed consent was obtained electronically, ensuring confidentiality and anonymity. The overall response rate was approximately 82%, after excluding invalid or incomplete submissions. Demographic characteristics of the study sample are presented in Table 1.

**Table 1:** Demographics of the study participants.

Variable	Frequency	Percent%
Gender		
Male	230	72%
Female	91	28%
Marital status		
Married	132	41%
Single	112	35%
Widowed/divorced	77	24%
Duration of displacement		
Less than three years	39	12%
Three to five years	96	30%
More than five years	186	58%
Total	321	100%

### 2.2 Instruments

#### 2.2.1 Mindfulness

The Five Facet Mindfulness Questionnaire (FFMQ), which comprises 39 items (Baer, 2007), has been demonstrated to be a reliable and valid scale in Arabic settings (Aldbyani, 2023). The Arabic version of the FFQM has five dimensions: Observing, Describing, Acting with Awareness, Non-reactivity, and Nonjudging. Each item was rated on a five-point Likert scale, with one indicating “never or very rarely true” and five indicating “very often or always true.” In this study, Cronbach’s alpha was 0.78.

#### 2.2.2 Distress tolerance

The Distress Tolerance Scale (DTS), which comprises 15 items (Simons and Gaher, 2005), has been demonstrated to be a reliable and valid scale in Arabic settings (e.g., Noureddine et al., 2025). Each item was rated on a five-point Likert scale—these terms ranged from 1 (*strongly disagree*) to 5 (*strongly agree*). In our study, Cronbach’s alpha was 0.81.

#### 2.2.3 Trauma recovery

The Posttraumatic Diagnostic Scale for DSM–5 (PDS-5), which contains 20 items (Foa et al., 2016), has been proven as a reliable and valid scale in Arabic settings (e.g., Alghamdi and Hunt, 2020). Each item was rated on a five-point Likert scale—these terms ranged from 1 (*strongly disagree*) to 5 (*strongly agree*). In our study, Cronbach’s alpha was 0.80.

### 2.3 Data analysis

Pearson’s correlation coefficients were calculated to examine the associations among the study variables. Mediation analyses were performed using the PROCESS macro for SPSS (version 3.5; Hayes, 2012). The significance of the indirect effects was evaluated using 95% bias-corrected confidence intervals based on 5,000 bootstrap resamples.

## 3 Results

### 3.1 Correlation among study variables

The findings, as presented in Table 2, indicate that all dimensions of mindfulness—observing, describing, acting with awareness, non-reactivity, and non-judgment—are positively correlated with both distress tolerance and trauma recovery among internally displaced individuals.

**Table 2:** Correlation among study variables.

Variables	1	2	3	4	5	6	7
1. Observing	1						
2. Describing	0.44**	1					
3. Acting with awareness	0.46**	0.45**	1				
4. Non-reactivity	0.57**	0.59**	0.56**	1			
5. Non-judgment	0.50**	0.42**	0.49**	0.45**	1		
6- Distress Tolerance	0.35*	0.43**	0.42**	0.38*	0.33*	1	
7- Trauma Recovery	0.38	0.34	0.40	0.36	0.38	0.75**	1

*p < 0.05, **p < 0.01.

### 3.2 Mediation effects

To explore the influence of mindfulness on trauma recovery, a bias-corrected percentile bootstrap method was employed using Model 4 in the SPSS PROCESS macro (5,000 resamples, 95% confidence interval). In this analysis, mindfulness served as the independent variable, trauma recovery as the dependent variable, and distress tolerance as the mediating variable. The results of the mediation analysis are summarized as follows:

#### 3.2.1 Observing

**Figure 1:**
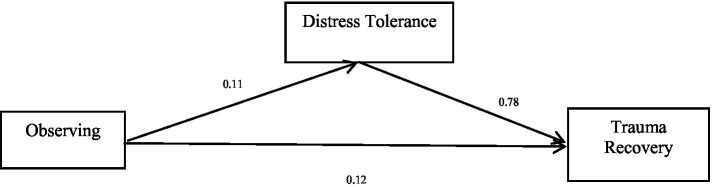
Mediation effect of observing on trauma recovery.

The total effect of the first dimension of mindfulness, observing, on trauma recovery is significant (*b* = 0.21, *p* < 0.05), and the direct effect of observing on trauma recovery is significant (*b* = 0.12, *p* < 0.05). Observing positively predicted trauma recovery. Distress tolerance positively predicted trauma recovery. Finally, the bias-corrected percentile bootstrap method indicated that the indirect effect of observing on trauma recovery through distress tolerance was significant, *ab* = 0.09, *SE* = 0.02, *p* < 0.05, 95% CI = [0.05, 0.12]. This indicates that distress tolerance partially mediated the effect of observing on trauma recovery. See Table 3 and Figure 1 for more details.

**Table 3:** Mediation effect of observing on trauma recovery.

Model	*b*	*SE*	95% CI
Observing → Trauma recovery (*a*)	0.11	0.02	[0.07, 0.14]
Distress tolerance → Trauma recovery (*b*)	0.78	0.04	[0.70, 0.85]
Observing → Trauma recovery (*c’*)	0.12	0.02	[0.08, 0.15]
Observing → Distress tolerance → Trauma recovery	0.09	0.02	[0.05, 0.12]
Total effect (Observing → Trauma recovery) (*c*)	0.21	0.02	[0.17, 0.24]

*b* = unstandardized regression coefficient; *SE* = standard error; CI = confidence interval.

#### 3.2.2 Describing

**Figure 2:**
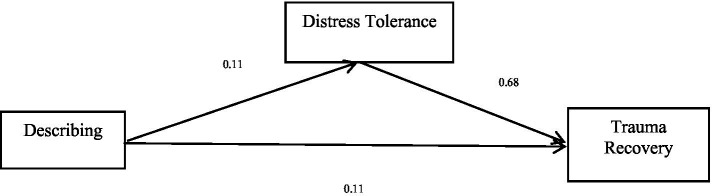
Mediation effect of describing on trauma recovery.

The total effect of the second dimension of mindfulness, describing trauma recovery, is significant (*b* = 0.18, *p* < 0.05), and the direct effect of describing trauma recovery is significant (*b* = 0.11, *p* < 0.05). Describing positively predicted trauma recovery. Distress tolerance positively predicted trauma recovery. Finally, the bias-corrected percentile bootstrap method indicated that the indirect effect of describing on trauma recovery through distress tolerance was significant, *ab* = 0.07, *SE* = 0.03, *p* < 0.05, 95% CI = [0.03, 0.10]. This indicates that distress tolerance partly mediated the effect of describing on trauma recovery. See Table 4 and Figure 2 for more details.

**Table 4:** Mediation effect of describing on trauma recovery.

Model	*b*	*SE*	95% CI
Describing → Trauma recovery (*a*)	0.11	0.02	[0.7, 0.14]
Distress tolerance→ Trauma recovery (*b*)	0.68	0.04	[0.60, 0.75]
Describing → Trauma recovery (*c’*)	0.11	0.02	[0.07, 0.14]
Describing → Distress tolerance→ Trauma recovery	0.07	0.02	[0.03, 0.10]
Total effect (Describing → Trauma recovery) (*c*)	0.18	0.03	[0.12, 0.23]

#### 3.2.3 Acting with awareness

**Figure 3:**
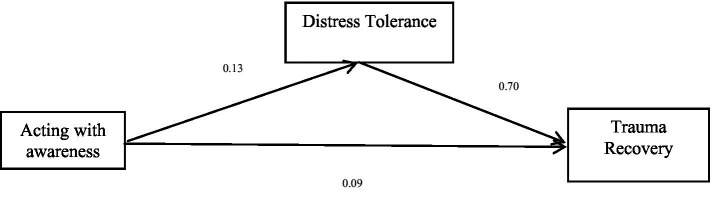
Mediation effect of acting with awareness on trauma recovery.

The total effect of the third dimension of mindfulness, acting with awareness on trauma recovery, is significant (*b* = 0.18, *p* < 0.05), and the direct effect of acting with awareness on trauma recovery is significant (*b* = 0.09, *p* < 0.05). Acting with awareness was positively associated with trauma recovery. Distress tolerance positively predicted trauma recovery. Finally, the bias-corrected percentile bootstrap method indicated that the indirect effect of acting with awareness on trauma recovery through distress tolerance was significant, *ab* = 0.09, *SE* = 0.02, *p* < 0.05, 95% CI = [0.05, 0.12]. This indicates that distress tolerance partly mediated the effect of acting with awareness on trauma recovery. See Table 5 and Figure 3 for more details.

**Table 5:** Mediation effect of acting with awareness on trauma recovery.

Model	*b*	*SE*	95% CI
Acting with awareness → Trauma recovery (*a*)	0.13	0.02	[0.09, 0.16]
Distress Tolerance→ Trauma recovery (*b*)	0.70	0.03	[0.64, 0.75]
Acting with awareness → Trauma recovery (*c’*)	0.09	0.02	[0.05, 0.12]
Acting with awareness → Distress tolerance→ Trauma recovery	0.09	0.02	[0.05, 0.12]
Total effect (Acting with awareness → Trauma recovery) (*c*)	0.18	0.03	[0.12, 0.23]

#### 3.2.4 Non-reactivity

**Figure 4:**
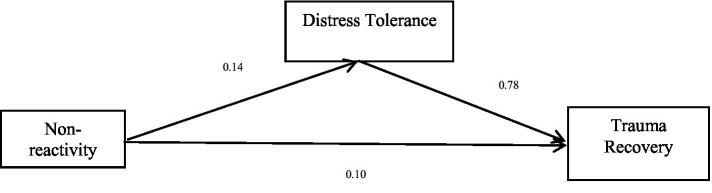
Mediation effect of non-reactivity on trauma recovery.

The total effect of the fourth dimension of mindfulness, non-reactivity, on trauma recovery is significant (*b* = 0.20, *p* < 0.05), and the direct effect of non-reactivity on trauma recovery is significant (*b* = 0.10, *p* < 0.05). Non-reactivity positively predicted trauma recovery. Distress tolerance positively predicted trauma recovery. Finally, the bias-corrected percentile bootstrap method indicated that the indirect effect of non-reactivity on trauma recovery through distress tolerance was significant, *ab* = 0.11, *SE* = 0.02, *p* < 0.05, 95% CI = [0.07, 0.14]. This indicates that distress tolerance partly mediated the effect of non-reactivity on trauma recovery. See Table 6 and Figure 4 for more details.

**Table 6:** Mediation effect of non-reactivity on trauma recovery.

Model	*b*	*SE*	95% CI
Non-reactivity → Trauma recovery (*a*)	0.14	0.02	[0.10, 0.17]
Distress Tolerance→ Trauma recovery (*b*)	0.78	0.04	[0.70, 0.85]
Non-reactivity → Trauma recovery (*c’*)	0.10	0.02	[0.06, 0.13]
Non-reactivity → Distress tolerance→ Trauma recovery	0.11	0.02	[0.07, 0.14]
Total effect (non-reactivity → Trauma recovery) (*c*)	0.20	0.03	[0.14, 0.25]

#### 3.2.5 Non-judgment

**Figure 5:**
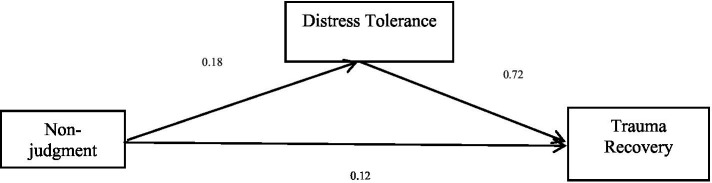
Mediation effect of non-judgment on trauma recovery.

The total effect of the fifth dimension of mindfulness, non-judgment, on trauma recovery is significant (*b* = 0.25, *p* < 0.05), and the direct effect of non-judgment on trauma recovery is significant (*b* = 0.12, *p* < 0.05). Non-judgment positively predicted trauma recovery. Distress tolerance positively predicted trauma recovery. Finally, the bias-corrected percentile bootstrap method indicated that the indirect effect of non-judgment on trauma recovery through distress tolerance was significant, *ab* = 0.13, *SE* = 0.02, *p* < 0.05, 95% CI = [0.09, 0.19]. This indicates that distress tolerance partly mediated the effect of non-judgment on trauma recovery. See Table 7 and Figure 5 for more details.

**Table 7:** Mediation effect of non-judgment on trauma recovery.

Model	*b*	*SE*	95% CI
Non-judgment → Trauma recovery (*a*)	0.18	0.02	[0.14, 0.21]
Distress Tolerance→ Trauma recovery (*b*)	0.72	0.04	[0.64, 0.79]
Non-judgment → Trauma recovery (*c’*)	0.12	0.02	[0.08, 0.15]
Non-judgment → Distress tolerance→ Trauma recovery	0.13	0.02	[0.09, 0.16]
Total effect (non-judgment → Trauma recovery) (*c*)	0.25	0.03	[0.19, 0.30]

## 4 Discussion

The first aim of our study was to investigate the relationship between mindfulness, distress tolerance, and trauma recovery among Yemeni internally displaced persons. Consistent with prior research (Elhai et al., 2018; Li et al., 2023; Zeifman et al., 2020), the findings indicate that mindfulness dimensions are significantly associated with distress tolerance and trauma recovery, suggesting that mindfulness may function as a protective resource for individuals exposed to displacement-related stress.

However, our study utilized trait (dispositional) mindfulness as measured by the FFMQ (five dimensions). To our knowledge, few studies have been conducted using FFMQ with these three variables together. These relationships between the study variables are because being mindful of these different dimensions can provide insight into how they are associated with distress tolerance, which in turn is associated with trauma-related recovery.

In predominantly Muslim contexts such as Yemen, mindfulness may be naturally embedded within daily religious practices. Ritual prayer (salat), remembrance (dhikr), and supplication (dua) emphasize attentiveness, present-moment awareness, and acceptance, which parallel core elements of secular mindfulness (Aldbyani and Al-Abyadh, 2023; Koenig, 2012; Aldbyani, 2025). Empirical studies have demonstrated that mindful prayer is associated with greater psychological well-being and lower distress (Ijaz et al., 2017), while dhikr-based interventions have been shown to reduce anxiety and enhance gratitude (Rahman et al., 2023). Similarly, integrating breathing techniques with dhikr has been shown to improve sleep quality and overall quality of life among clinical populations (Purwanto et al., 2023). These findings suggest that culturally embedded practices provide meaningful avenues for enhancing resilience and recovery in Muslim populations. In the context of Yemeni internally displaced persons, acknowledging these practices enriches the cross-cultural contribution of the study and suggests the potential of culturally congruent interventions for trauma recovery.

The second aim of our study was to investigate the potential role of distress tolerance as a mediator in the association between mindfulness dimensions and trauma recovery. Distress tolerance was identified as a plausible pathway linking mindfulness to trauma-related outcomes. This implies that the benefits of mindfulness are partially realized through its capacity to enhance individuals’ ability to withstand and regulate distress, which subsequently facilitates recovery after trauma.

The positive association found between all mindfulness facets and distress tolerance can be explained by the core mechanism of mindfulness: enhancing awareness of thoughts and emotions without judgment. This process, often referred to as decentering (Thompson et al., 2011), likely contributes to the improved emotional regulation and distress tolerance observed in our sample, which in turn facilitated their trauma recovery.

Although the present study consistently identified positive associations among mindfulness, distress tolerance, and trauma recovery, it is essential to acknowledge that not all individuals may benefit equally from mindfulness practices. Some participants reported relatively low levels of mindfulness and distress tolerance, which suggests that aggregate analyses may not fully capture variability in these constructs. Moreover, prior research indicates that mindfulness can be challenging for specific individuals, particularly in contexts of high trauma exposure, where increased awareness of distressing thoughts may initially heighten discomfort (Britton, 2019). Recognizing this variability emphasises the need for future research to explore null or adverse responses, as well as potential moderators (e.g., trauma severity, coping style), to provide a more balanced understanding of the role of mindfulness in trauma recovery.

This study has several limitations. First, the sample was heavily male-dominated (72%), which limits the generalizability of the results to women and more diverse demographic groups. Gender dynamics may influence both mindfulness and trauma recovery, and future research should aim for more balanced samples to capture these differences better. Second, participants were recruited online through social media platforms, which raises concerns about representativeness and introduces the possibility of self-selection bias; individuals with internet access and interest in the study topic may differ systematically from those who did not participate. Third, the study employed a cross-sectional design, which restricts the ability to draw causal inferences about the directionality of the observed relationships. Longitudinal or experimental designs would be needed to confirm causal pathways. Finally, reliance on self-report measures increases the risk of common-method variance and social desirability bias. Future research should therefore employ multi-method approaches, such as clinical interviews or behavioral indicators, to reduce shared method bias and strengthen the validity of the findings.

## 5 Conclusion

This study investigated the relationship between mindfulness and trauma recovery within internally displaced individuals, focusing on the potential mediating role of distress tolerance. The results indicated that the five facets of mindfulness—observing, describing, acting with awareness, non-reactivity, and non-judgment—were positively associated with both trauma recovery and distress tolerance.

The findings revealed that distress tolerance partially mediated the relationship between each facet of mindfulness and trauma recovery, indicating that individuals with higher levels of mindfulness exhibited greater capacity to tolerate psychological distress, which in turn enhanced their recovery from trauma. Mediation models provided evidence consistent with a mediation pathway involving distress tolerance.

The present findings contribute to a growing body of evidence supporting the role of mindfulness as a protective psychological resource, specifically identifying distress tolerance as a key mechanism in a high-adversity, displacement-affected population. In the context of internally displaced persons in Yemen, where individuals are exposed to chronic stressors and limited mental health resources, fostering mindfulness and distress tolerance may represent a potentially feasible and culturally appropriate approach.

These findings may inform trauma recovery programs regarding the inclusion of mindfulness and distress to foster emotional regulation, psychological flexibility, and resilience. Future research should aim to replicate these findings in longitudinal and intervention-based designs, as well as across diverse cultural and displacement contexts, to validate further the causal pathways and practical implications identified in this study.

## Data Availability

The raw data supporting the conclusions of this article will be made available by the authors, without undue reservation.
